# Exploring preferences to accessing sexual and reproductive health services: A qualitative study of adolescents’ and service provider perspectives

**DOI:** 10.1371/journal.pone.0312872

**Published:** 2024-12-04

**Authors:** Negussie Boti Sidamo, Amene Abebe Kerbo, Kassa Daka Gidebo, Yohannes Dibaba Wado

**Affiliations:** 1 School of Public Health, College of Health Sciences and Medicine, Wolaita Sodo University, Wolaita, Sodo, Ethiopia; 2 School of Public Health, College of Medicine and Health Sciences, Arba Minch University, Arba Minch, Ethiopia; 3 African Populations and Health Research Center, Nairobi, Kenya; Aga Khan University pakistan, PAKISTAN

## Abstract

**Background:**

Understanding what adolescents want and how preferences are likely to vary among different groups is important to make the healthcare system responsive to the needs of adolescents and to maximize service utilization. Despite this, evidence is scarce in this aspect. Therefore, this study aimed to explore preferences for accessing Sexual and Reproductive Health (SRH) services from the perspective of adolescents and health care providers in the South Ethiopia Regional State.

**Methods:**

A phenomenological qualitative study was conducted from September 04 to October 15, 2023. Seven Focus Group Discussions (FGDs) and ten Key Informant Interviews (KIIs) were conducted with purposively selected seventy-five adolescents and ten healthcare providers respectively. A semi-structured interview guide was used to explore their lived experiences. All interviews and discussions were audio-recorded. The research team transcribed the collected data verbatim and translated it into English. The data was analyzed using a thematic analysis approach.

**Results:**

In this study, five main themes were identified. The identified main themes were preferred SRH services provider, preferred SRH services venue, preferred SRH services provision time, preferred SRH services information sources, and preferred strategies to improve access to SRH services. Regarding preferred SRH service providers, adolescents prefer males by sex, younger health care providers by age, and not neighborhood health providers by residence. Concerning preferred SRH services venue, adolescents prefer pharmacies as a venue to access condoms and emergency pills, while they prefer traditional medical centers as a source of abortion services. Besides this, schools are a preferred venue for adolescents to access SRH information. Moreover, sexual partners, close friends, and peers who experienced similar problems are the preferred sources of SRH information for adolescents. Furthermore, engagement of private health facilities, strengthening engagement of faith-based organization, and strengthening community-based SRH services provision were identified as preferred strategies to improve access to SRH services.

**Conclusion:**

Improving access to sexual and reproductive health services necessitates the implementation of innovative and responsive strategies that address the diverse preferences and needs of adolescents. Prioritizing adolescent engagement in healthcare is vital, as it fosters a better understanding of their unique perspectives and ultimately enhances the accessibility and utilization of these essential services.

## Background

Globally, Sexual and Reproductive Health (SRH) problems persist as a major public health concern [1]. Adolescents (10–19 years) disproportionately bear the burden of SRH problems [2,3]. In low-income countries like Ethiopia, adolescents were struggling with high mortality from preventable causes of SRH problems [4]. Studies also show that adolescent is at a higher risk of acquiring sexually transmitted infections (STIs) due to their high-risk sexual behaviors, including unprotected sex [5–7].

Establishing healthy sexual behaviors and pathways during adolescence can have a positive impact on one’s well-being in later adulthood [8]. Evidence shows that early interventions, which can be more effective in adolescence than in adulthood, can prevent delayed harm [9,10]. These significant segments of the population’s growing needs and access to SRH services had not been sufficiently addressed [11–13]. Additional research finding indicates that adolescents express explicit opinions regarding the provision of healthcare services delivery [9,14,15].

The current WHO recommends a health systems approach that is responsive to the diverse needs and preferences of adolescents [16]. The term “adolescent-responsive health systems approach” refers to a move away from stand-alone youth-friendly service models towards strengthening the different components of a health system to meet the different needs and to better meet the preferences of adolescent [16]. It may also mean that every component of the health system, including the public and private sectors and communities, should be designed to address the health needs of adolescent in a way that is responsive to their needs [16].

In Ethiopia, there has been increased attention to YFS services [17–20]. Nevertheless, several obstacles, including low use of specialized services and staff and resource shortages, have made it difficult to sustain these programs [21,22]. Additionally, data demonstrates that most adolescent-friendly rooms in medical facilities are crammed with dusty boxes of supplies after donor funding ends [23]. Many young people are left perplexed by this narrow approach as to where they are welcome in health facilities and, in certain cases, what services they can access [23]. There are still millions of adolescents who lack access to any health care, and SRH services in particular. This has worsened in the context of the COVID-19 pandemic [21].

Understanding the adolescents’ preferences plays a critical role in improving access to and utilization of SRH services [8,15,24]. To optimally utilize SRH services, the healthcare system needs to be responsive to the expectations of adolescents it serves [15,25,26]. To make the healthcare system responsive, it is important to understand what adolescents want and how preferences are likely to vary among different groups [15]. The results of earlier research emphasized the significance of taking adolescents’ preferences for SRH service access into account [8,26]. In addition, researching adolescents’ preferences is vital for understanding determinants of healthcare demand and designing effective health promotion and early intervention strategies [9]. On the other hand, failing to recognize the preferences of adolescents can lead to the wastage of resources within the healthcare system [27,28]. Therefore, this study aimed to explore preferences to access SRH services in the Gamo zone, south Ethiopia from adolescents’ and healthcare providers’ perspectives.

## Methods and materials

### Study setting and period

This study was conducted in the *Gamo zone*, which is located in the southern regional state of Ethiopia. Administratively, Ethiopia is classified into four levels: the first level (*regions*), the second (*zones*), the third (*woredas*), and the fourth level (*kebeles)*. *Kebeles is* the lowest administrative unit with 3,000 to 5,000 inhabitants [29]. The *Gamo zone* borders the *South Omo* zone to the southwest, the Wolayta and Gofa zones to the north, the Amaro and Dirashe special woreda to the southeast, and the Lake Abaya to the northeast. Arba Minch town is the administrative center of this Zone. This town is located 431 km from the Ethiopian capital city (Addis Ababa). Six town administrations and 14 rural districts with 306 *kebeles* were found in the Gamo zone. The total population in this zone is 1,643,205 of those 805,205 are male and 838,034 female [30] There are currently 363 public health facilities providing preventive and curative services to the community. Of these, five are primary hospitals, one general hospital, 59 health centers, and 297 health posts. In addition, there are 251 private healthcare facilities. Of these, one primary hospital, 190 private clinics, 56 private pharmacies, and four drug stores.

According to the 2023 performance report of the Health Department of Gamo Zone (unpublished), family planning coverage was 78%, of which 6.09% were adolescents aged 10 to 19 years. HIV testing coverage was 75%, antenatal care (ANC) coverage was 98%, and institutional delivery coverage was 73%. This study was conducted from September 4 to October 15, 2023.

### Study design

A qualitative phenomenological approach was chosen as the research methodology to address the research question: "How do adolescents prefer to access sexual and reproductive health services?" This approach is particularly appropriate for exploring phenomena from the participants’ perspectives, allowing for an in-depth understanding of their lived experiences.

Phenomenology focuses on capturing the essence of these experiences, providing valuable insights into adolescents’ perceptions and preferences regarding SRH services. By uncovering the nuanced and often hidden aspects of their experiences, this methodology facilitates a comprehensive understanding of the factors influencing how adolescents navigate access to these essential services.

Qualitative methodologies are particularly recommended for investigating sensitive topics such as sexual and reproductive health, as they enable researchers to delve into the complexities of individuals’ life experiences and perceptions. By employing a phenomenological approach, this study aims to reveal the unique challenges and motivations adolescents face when accessing SRH services. The insights gained from this research will be beneficial for various stakeholders, including adolescents themselves, their parents or caregivers, healthcare providers, health service managers, and policymakers. Thus, the selection of a phenomenological approach is justified as it allows for a rich exploration of adolescents’ subjective realities, ultimately informing the development of more tailored and effective SRH services that resonate with their specific needs and preferences.

*Target Population*. All service users (adolescents) who live in the selected study area and all service providers (health care providers) working in selected health facilities during the study period and fulfilling the eligibility criteria were the target population.

*Eligibility Criteria*. Those adolescents aged 10 to 19 years living in the study area were included as service users, as well as service providers who provided SRH services to adolescents in the selected public health facility during the study period.

### Sample size for qualitative study

Generally, for a qualitative study, there is no pre-determined method for determining the ’required’ sample size. However, the sample size for the qualitative section is determined based on information saturation at a point where new information is no longer forthcoming. Information saturation is achieved when there are a sufficient number of participants to be able to identify the full range of views and understandings regarding various components of the study. Thus, the number of FGDs and KIIs was determined by the point of information saturation [31,32].

*Participants’ selection*. A purposive sampling technique was used to select the target study participants. Hence purposive sampling is appropriate when you want to recruit people who have had a specific experience or been exposed to a particular phenomenon, you will intentionally or purposefully be reaching out to people that you know have had this experience. Thus, adolescents who had experiences with SRH services and healthcare providers who were directly involved in the provision of SRH services for adolescents were selected using the purposive sampling technique due to their lived experience with the area of interest and detailed information about the phenomenon under investigation. This helps us to get rich and thick information about the phenomenon of interest. Community health extension workers facilitate the selection of adolescents. A total of seven FGDs, including 9–14 participants per group with adolescents. In addition, ten SRH service providers were involved in key informant interviews (KIIs). Focus group discussions have an average duration of 61 minutes with a maximum of 75 minutes and a minimum duration of 48 minutes. Key informant interviews have an average duration of 41 minutes with a maximum duration of 47 minutes and a minimum duration of 36 minutes.

### Data collection procedure

Key informant interviews (KII) and focus group discussions (FGD) were used to gather the qualitative data. Interview guides were prepared based on based on previously published and conducted similar studies [33–37]. The data collection tool was initially created in English and then translated into Amharic. The interview was conducted in Amharic. The main data collection process was started after explaining the aim of the study, reading the consent form to each respondent, and asking them to participate in the study. Once their consent was obtained, the interview date and place of the interview were arranged in advance with each respondent. FGDs with adolescents were the first step in the data collection process. A detailed interview guide is available **“S1 Appendix”.**

These FGDs were conducted at health centers, health posts, and youth-friendly centers. No one, including the staff of the health post, health center, and youth-friendly center, is allowed to access the discussion area. Adolescent groups were stratified into two homogenous categories based on their sex. Group 1: comprised of adolescent girls aged 10 to 19 years. Group 2: comprised of adolescent boys aged 10 to 19 years. The discussions of adolescents were facilitated by the same sex. The FGDs with boys group was facilitated by the principal investigator and two trained research assistants, while the discussion of girls was facilitated by a trained female moderator and two trained research assistants.

Furthermore, KIIs were conducted with the SRH service providers within the selected study area. The interviews were conducted at the participants’ respective workplaces to maintain privacy as well as convenience. All FGDs and KIIs were held in the Amharic language. It was recorded using a digital tape recorder with prior permission from the study participants. A total of three experienced qualitative data collectors participated in the data collection. One of the research assistants served as a moderator, and the second actively took notes. The note taker, takes notes during discussion and interview after obtaining permission from the study participants. These handwritten notes helped to supplement audio-recorded transcript information. At the end of each discussion and interview, the research assistants went over the main points raised and confirmed with the discussants that their points had been captured accurately before ending each interview session. Each day the principal investigators and research assistants held a debriefing session to reflect on the conducted discussions regarding the main issues raised as well as areas that brought challenges and insights.

### Qualitative data analysis

All interviews and discussions were audio-recoded. The recorded data were transcribed verbatim into the local language by the same research assistants who conducted the interviews and discussions. Then it was translated into English for analysis. Research teams independently reviewed the transcripts. The data were analyzed through thematic analysis. Major themes were derived based on the objective of the study. However, subthemes were induced from the text itself through repeated reading. Transcripts were coded using ATLAS Ti version 7 software. Statements were coded and arranged according to the related theme. The transcripts were reread after themes were determined to make sure the themes accurately represented the data’s content. All of the themes were thought to adequately represent the conversations from the KIIs and FGDs. The findings were presented in narratives by thematic areas based on the objectives of the study. The quotes included in the results were typical views expressed in each KII/FGD to exemplify the emergent theme.

### Trustworthiness of the study

To establish the trustworthiness of this qualitative research, we systematically implemented the key concepts of credibility, dependability, transferability, and confirmability. We enhanced credibility through both data source and methodological triangulation. Data source triangulation involved engaging diverse participant groups, including service users and providers, while methodological triangulation incorporated focus group discussions (FGDs) and key informant interviews (KIIs). Additionally, we conducted member checks by returning each transcript to participants, allowing them to identify and rectify any inaccuracies or misinterpretations.

The research team brought significant prior experience in qualitative research and a comprehensive understanding of the community’s cultural context, fostering rapport and encouraging participants to provide open and honest responses. We utilized purposive sampling to select individuals with direct experience relevant to the phenomena under investigation, thereby maximizing the credibility of our findings. Probing interview techniques facilitated deeper exploration of participants’ perspectives, supported by audio recordings and detailed written field notes that captured nonverbal cues. Daily peer debriefing sessions further promoted reflective analysis and verification of the data.

To ensure dependability, we maintained an audit trail involving both the supervisor and co-supervisor, which confirmed that our analyses were consistently aligned with the data. Rigorous standards were upheld throughout the study, with interviews continuing until data saturation was achieved. For transferability, we provided comprehensive descriptions of the study context and results, while including verbatim quotations in our reporting to allow readers to evaluate the applicability of our findings to other contexts.

To promote confirmability and mitigate investigator bias, the principal investigator consciously set aside personal assumptions, perceptions, and prior knowledge during data collection, coding, and analysis. Reflexive practices were employed, including the use of reflexive journals, field notes, and memos, to validate the data collected. Throughout the interpretation of findings, we ensured a careful balance between our interpretations and direct quotations from study participants, preserving the authenticity of their voices. Importantly, the context of this study is distinct from the environments in which the principal investigators typically work and reside, further contributing to the integrity of the findings. By meticulously addressing these dimensions of trustworthiness, we aim to establish a robust foundation for our study’s findings, ensuring they are credible, dependable, transferable, and confirmable.

## Ethical considerations

Ethical clearance was obtained from the Institutional Research Ethics Review Committee (IRRC) of Wolaita Sodo University on February 9, 2023 (project reference number: WSU-IRRC/004/2023). Before the fieldwork, necessary communications about the overall purpose of the study were made with the respective responsible bodies. A formal letter of permission was obtained from the Gamo Zone health department. Written informed consent was obtained from all study participants after clearly describing the purpose of the study, benefits, and risk of participation, anonymity and the right to refuse at any stage of the interview. For participants under 18 years old, assent was obtained from study participants and written Informed consent was obtained from their parent and/or legal guardian. Participation of the participants in the study was voluntary. There was an opportunity to ask questions about the study and the right to decline or cancel the interview. Privacy and confidentiality of information of the study participants were assured before obtaining data. About confidentiality, respondents were given information that guaranteed them that the information they provided during the study would be used for the research purpose and would not be disclosed to anybody outside the research team. All methods were followed according to the Helsinki Declaration.

## Results

### Characteristics of study participants

Ten key informant interviews (KIIs) and seven focus group discussions (FGDs) were conducted in the South Ethiopia regional state. A total of 75 individuals participated in seven FGDs and 10 key informants participated in the study. The mean age of the focus group participants was 15.61 (SD ±2.31) years, ranging from 11 to 19 years. Whereas the mean age of the key informants was 34.40 (SD ±7.25) years, ranging from 30 to 54 years. Slightly more than half (60%) of the focus group discussants were female. The mean year of key informants’ work experiences was 12 (SD ±7) years, ranging from 6 to 30 years. Details on these study participants are available in **“S2 Appendix”.**

## Themes and subthemes

In this study, five main themes were identified. The identified main themes were preferred SRH services provider, preferred SRH services venue, preferred SRH services provision time, preferred SRH services information sources, and preferred strategies to improve access to SRH services **(Table 1).**

**Table 1:** Main themes and sub-themes identified in this study.

Number of themes	Main themes	Subthemes
**Theme 1**	Preferred SRH services provider	Preferences by sex of service provider
		Preferences by age of service provider
		Preferences by residences of service provider
**Theme 2**	Preferred SRH services venue	Pharmacies as the preferred venue for adolescents
		School as a preferred venue for adolescents
		Traditional herbal centers as the preferred venue for adolescents
		Private health facilities as the preferred venue for adolescents
		Governmental health facilities as the preferred venue for adolescents
**Theme 3**	Preferred SRH services provision time	Regular working time
		Extended working time
**Theme 4**	Preferred SRH services information sources	Sexual partners as preferred sources of information
		Peers who experienced similar problems as preferred sources of information
		Parents as preferred sources of information
		Healthcare providers as preferred sources of information
**Theme 5**	Preferred strategies to improve access to SRH services	Engagement of Private health facilities in SRH services provision
		Strengthen engagement of faith-based organization
		Strengthen community-based SRH services provision

## Theme 1: Preferred SRH services provider

The first main theme identified in this study is the preferred providers of SRH services. Understanding adolescents’ preferences is crucial for improving service delivery and ensuring that health professionals meet the specific needs of this population. This theme encompasses three subthemes: preferences based on the sex of the provider, preferences based on the age of the provider, and preferences based on the provider’s residence.

### Subtheme: Preferences based on the sex of the healthcare provider

A significant number of focus group participants indicated a preference for male health professionals for SRH services. Previous research suggests that adolescents may feel more comfortable discussing sensitive topics with male providers, particularly in cultures where gender norms influence healthcare interactions. Participants cited the calm demeanor of male providers, contrasting it with perceived anger and gossip among female providers.

“*I would prefer if the health professional is male. When the health professional is female, she wills rumors to everyone, and they get angry.*”[**17 years old female discussant]**

“*It is easier for me when it’s a male healthcare provider*. *Female healthcare providers get angry and hateful*. *But*, *male professionals ask calmly”* [**15 years old female discussant]**

“*Male healthcare provider*! *I can speak clearly with male healthcare providers”* [**18 years old female discussant]**

“*Since I am a boy*, *I prefer male health professionals*.*"* [**14 years old male discussant]**

“*If the health provider is male*, *I am not afraid to ask him*.*”* [**13 years old male discussant]**

Healthcare providers echoed this preference, with one male provider sharing;

“*I serviced here* [health center] *for the last ten years, and what I have seen during this time is, that female health professionals after they provide services for adolescents most of them speak loudly about the patients for the other teams. While the patient is hearing. While they are speaking the adolescents will hear what they are speaking. The adolescents will go back to their homes. They assume that their information is also disseminated like this. They never want like this. I am seeing such a thing in our facility. So, most adolescents do not trust female health professionals".* **[30-year-old Male, Key informant].**

Another female healthcare provider noted:

“*Some adolescents prefer male healthcare providers. For instance, when they come with complaints of sexually transmitted infection, despite my presence, they will ask me to bring a male care provider. I think they prefer male healthcare providers to treat them”* **[37 years old female, Key informant]**

Conversely, some participants expressed a preference for female providers, citing empathy and compassionate care. Research indicates that female providers can offer unique perspectives and support, enhancing adolescents’ comfort and satisfaction with care.

“*I would prefer a female healthcare provider because she can teach me from her own experience.*” [**16 years old female discussant]**

“*I prefer female health professionals because females are secret keepers*. *However*, *male health professionals will talk with the father and brother*.*”* [**16 years old female discussant]**

“*I think it would be preferable if the health professional is female*.” [**17 years old female discussant]**

Female healthcare providers also advocated for female professionals in adolescent care:

"*I prefer female service providers should be given those services for adolescents. Because male providers can invite girls into unnecessary relationships. Females can show empathy and compassionately provide care"* **(30 years old female, Key informant]**

“*I think the preferred service provider for adolescents will be a female healthcare provider because they can keep confidentiality*.” **[31 years old female, Key informant]**

However, a male provider acknowledged the prevailing preference for male providers among youth:

“I *think it would be good if the service provider for young people is a female health professional, but young people prefer a male health professional to a female health professional.”* ***(*31 years old Male, Key informant]**

Key informants and discussants emphasized that preferences are context-dependent, suggesting that male providers are preferable for boys and female providers for girls. This aligns with existing literature that indicates the importance of gender matching in healthcare settings.

“*For boys, it is better if the health provider is male. For girls, it is better if the health provider is female.*” [**12 years old male discussant]**

“*If the health provider is male for adolescent girls*, *they may think of something else sexual when they provide services*.” [**13 years old male discussant]**

“*Any health professional that knows prefers to give services to adolescents*.*"* [**15 years old male discussant]**

### Subtheme: Preferences based on the age of the healthcare provider

Regarding the age of healthcare providers, younger adolescents tended to prefer older service providers, likely due to perceived maturity and experience. In contrast, older adolescents favored younger healthcare workers who were closer in age, as this may facilitate a more relatable and comfortable interaction. This age dynamic highlights the importance of matching healthcare providers with adolescents to promote effective communication and trust.

“*Those who are higher than us should learn first then teach us about sexual and reproductive health issues.”* [**12 years old Male discussant]**

“Older *health professionals should prefer services provider for me to give sexual and reproductive health services*.*”* [**14 years old female discussant]**

The preference of older adolescents for younger healthcare workers can be attributed to several factors that enhance their comfort in accessing sexual and reproductive health services. While younger adolescents tend to favor older providers for their perceived experience, older adolescents feel that peers closer in age can foster a more open dialogue.

One female discussant articulated, *“Those sexual and reproductive health service providers should be preferred if they are older health professionals; the younger health professionals are not approachable.”* Another emphasized, *“Even if they know about the services, they may not utilize them because older providers make it difficult to seek help with confidence.”* A male discussant affirmed, *“The younger health professionals prefer to provide sexual and reproductive health services for us!”*

These findings highlight the importance of age alignment in establishing trust and facilitating effective communication, echoing the earlier theme regarding preferences based on provider age.

In this theme, "Preferences by Residences of the Service Provider," most focus group discussants indicated a strong preference for healthcare providers who are unfamiliar to them, as opposed to neighborhood providers. They expressed concerns regarding confidentiality, fearing that local healthcare workers might disclose sensitive information to their families.

For instance, one female discussant stated, *“If the healthcare provider is from our area*, *I will return*. *Because they know my family*, *they may tell about me*.*”* This quote illustrates the adolescents’ heightened anxiety about potential breaches of confidentiality, linking to the earlier theme of "preferred SRH service providers," where comfort and trust were essential for open discussions about sensitive topics.

Similarly, a male discussant mentioned, “*It is better if I do not know the service provider because they [unfamiliar providers] hide my information*.*”* This highlights the perceived need for anonymity, suggesting that adolescents are more likely to seek care when they feel their privacy is safeguarded. This resonates with the previous theme, which noted that adolescents preferred male healthcare providers due to perceived calmness and discretion.

Key informants corroborated these concerns, noting that adolescents frequently avoid familiar healthcare providers because of the fear of exposure. One key informant stated, *“Most of the time*, *adolescents cannot freely access services because they fear to ask and they turn back if they know me*.*”* This reinforces the theme of confidentiality and the barriers it creates, further illustrating the adolescents’ preference for service providers who are not part of their immediate social circles.

Another key informant added, “*There are a lot of individuals who come to this health center from other areas to hide themselves from their relatives*…*because some providers may break the confidentiality of information*.*”* This comment underscores the critical role that perceived discretion plays in facilitating access to SRH services, linking back to the theme of preferred providers and their age and gender. Overall, the adolescents’ strong preference for unfamiliar healthcare providers emphasizes the intersection of confidentiality, comfort, and trust in their experiences accessing sexual and reproductive health services.

## Theme 2: Preferences for a venue for SRH services

### Subtheme: Pharmacies as the preferred venue for adolescents

The preferred places to access condoms and emergency pills are pharmacies and patent medicine vendor shops rather than public health facilities. Adolescents find it easier to express themselves to pharmacies and patent medicine vendors shop than health workers at health facilities. Also, adolescents feel access to service at such places is less stressful compared to the public health facilities.

“*Now we can get a condom from private drug stores and some shops for a fee”.* [**17 years old Male discussant]**

"*I will go to the pharmacy and ask for the post-pill*.*"* [***11 years old female discussant]***

“*Adolescents will go to private clinics to access services*.*”* ***(*32 years old Male, Key informant**]

### Subtheme: School as a preferred venue for adolescents

Most focus group discussants and key informants describe the school as the preferred place for adolescents to access SRH information. They explain that most sexual relationships start at school and most adolescents spend their time at school. So, school needed to be prioritized area to adolescents, but less attention was given by this time.

“*Strengthening school clubs is the easy and preferred method to teach adolescents about sexual and reproductive health issues.”* [**16 years old female discussant]**

“*Strengthening school clubs*, *then giving education through those clubs*, *because most love relationships start at school*. *So*, *such areas need to be prioritized for disseminating information*. *“[****18 years old female discussant]***

“*One approach is through school clubs*. *Even by this time*, *much attention is not provided for school clubs*.*”* **[32 years old Male, Key informant**]

“*……*. *when we think about young people*, *thinking about schooling is important*. *Therefore*, *we teach health education to young people in schools*. *I think schools are best for young people to fulfill their needs*. *“***[30 years old Female, Key informant]**

### Subtheme: Traditional herbal centers as the preferred venue for adolescents

The most preferred source of abortion services is traditional herbal centers or traditionalists such as local herb sellers, herbalists, and traditional birth attendants. The main reason given by the participants for their preferences is confidentiality of services provision in the traditional herbal centers, trust of services provision, as well as accessibility and affordability of such services in their local area.

“*This might be to keep their information confidential, as most assume that there is information confidentiality in traditional medical centers.”* [**18 years old Male discussant]**

“This *might be because traditional medical centers keep their patients’ information*. *They fear not to be prosecuted*!*”* [**15 years old female discussant]**

“T*hose adolescents from rich families will go to another area to get abortion services*, *but those from poor families will go to traditional herbs because for traditional herbs the payment is cheap*.*”* [**17 years old female discussant]**

"*If pregnancy happens most adolescent girls in our area will go to traditional medical centers with their trusted colleagues*.*"* [**16 years old Male discussant]**

### Subtheme: Private clinics as the preferred venue for adolescents

“*If my family has money, they could hide my pregnancy from our neighbors and they may take me to a far-away private clinic to abort the pregnancy.*” [**17 years old female discussant]**

“*Most adolescents prefer to access sexual and reproductive health services from private clinics because most adolescents easier to hide themselves from people than public health facilities*, *so many adolescents prefer to go there*.*”* **[30 years old Female, Key informant]**

### Subtheme: Governmental health facilities as the preferred venue for adolescents

Primary health centers were identified as an unpopular source to access abortion services for adolescents. Most participants explain that in most government health facilities there is no SRH services provision, most healthcare providers ask adolescents about parent or guardian consent or they neglect to provide sexual and reproductive health services.

“*Those* [sexual and reproductive health] *services are not provided in most government health facilities. Besides this, the cost of services is expensive. The health providers are not volunteers to provide services.”* [**11 years old female discussant]**

*“When we go to our nearby health facility*, *they will ask us about parent or guardian consent to access sexual and reproductive health services*. “[**18 years old female discussant]**

“*The healthcare providers neglect to provide sexual and reproductive health services for adolescents*.” [**18 years old male discussant]**

Healthcare providers, also confirm that due to the unavailability of abortion services in public health facilities, most adolescent girls go to traditional herbal centers.

“*In our area most girls when an unwanted pregnancy occurs, to get abortion services will go to traditional healers this is may be due to abortion services was not given in our facilities.”* **[31 years old female, Key informant]**

### Theme 3: Preferences based on service provision time

All Key informants said that to make health facilities responsive for adolescents there should not be a limit on service provision time. They explain that most adolescents come in the evening and on weekends.

“*Services provision of sexual and reproductive health needs to be 24-hour open. But, our health facility provides services at regular times only. No services provision by weekend and evening time.”* **[31 years old female, Key informant]**

"*The service should be provided 24 hours*. *Because many young people come in the evening for condoms and other family planning methods*. *But the room is closed in the evening without any service for them*.*"* ***[*30 years old Male, Key informant]**

“*In our institution*, *we provide the service until two o’clock in the evening*, *but mostly young people use it after two o’clock in the evening*.*”* ***[*31 years old Male, Key informant]**

“*……*.*Commercial sex workers often search for condoms at night”*. ***(*30 years old Male, Key informant)**

## Theme 4: Preferred SRH information sources for Adolescent

### Subthemes: Sexual partners as preferred sources of information

Most adolescents describe that their sexual partners are the preferred sources of SRH information

“*I will ask my boyfriend to bring me emergency pills, he will bring me.”* [**11 years old female discussant]**

“*I am sure my boyfriend will bring me a post-pill once I asked him to bring it because he does not want my pregnancy*. *I am thinking about the future before the child so that he can have such a world*. *I’m sure he doesn’t want the child; he’ll bring the medicine*.*"* [**11 years old female discussant]**

"*I will go with my girl’s friends to health center consult healthcare providers"* [**15 years old male discussant]**

“*I also will ask my boyfriend to bring me emergency pills; he will bring me because he does not want to be humiliated*.*”* [**17 years old female discussant]**

### Subthemes: Peers who experienced similar problems as preferred sources of information

Some focus group discussants describe that close friends, and peers who experienced similar problems are the preferred sources of SRH information for adolescents.

“*I will consult my close peers. If I know where I can get services, I will go. But most adolescents fear going alone."* [**17 years old female discussant]**

“*Before going to the health center*, *most girls initially advise their close friends*, *and then they go to the place they recommended*.*”*[**18 years old female discussant]**

“*……It is necessary to tell our problems to our friends when one of our friends might face problems because your friends may help you in searching solution for something like this*. *There is also a situation where people look down on you”* [**17 years old male discussant]**

“*I will consult with others who experienced sexual and reproductive health problems*, *especially commercial sex workers because they are vulnerable most of the time to such problems*.” [**16 years old female discussant]**

### Subthemes: Parents and Healthcare providers as preferred sources of information

Few of them also prefer to seek help from their parents and healthcare providers.

“*If I told my family about my love relationship when I started. I can tell them everything that happens after my love relationship. However, if I get pregnant without informing them about my relationship, they will fire me.”* [**17 years old female discussant]**

"*I would prefer to go to a health facility to discuss sexual and reproductive health"* [**18 years old female discussant]**

## Theme 5: Preferred strategies to improve access to SRH services

In this section, we present participants preferred strategies to improve access to SRH information and services for adolescents. The suggested prioritized strategies include engagement of private health clinics and engagement of religious organizations.

### Subthemes: Engagement of private health facilities in SRH services provision

All key informants do not support the engagement of private health facilities in the provision of sexual and reproductive health services for adolescents. They explain that the main focus of private clinics is how to collect money not to serve their community. Those facilities will provide those services without payment for adolescents.

“*The provision of sexual and reproductive health services provision is free of payment. When we see the provision of the services in private clinics is with payment. They will not give services free of payment. So to control such discrepancy it is difficult because we do not have policy and implementation guidelines to control.”* **[35 years old Male, Key informant]**

“*I do not think so*, *because the main focus of private clinics is how to collect money*. *I do not think they will provide those services without payment*. *Rather than expanding the provision of services to private clinics*, *correcting the current problems in most governmental facilities is crucial*.*”* ***[*32 years old Male, Key informant**]

“*Instead of providing free services to youth*, *private institutions are focused on money*. *I don’t think they provide free services to youth*. *In public institutions*, *those services are provided free of charge*, *but in private institutions*, *those services are provided by payment*.*”* ***[*31 years old Male, Key informant]**

“*I do not think so*! *In most private clinics there is only one health professional*, *but in public health facilities*, *there is high a professional composition*.*”* **[31 years old female, Key informant]**

### Subthemes: Strengthen engagement of faith-based organization

Key informants and focus group discussants describe that the religious leaders were role models of the community and adolescents can accept and apply what they said.

“*I think it would be easier to educate about sexual and reproductive health issues for young people in religious institutions. So, the government needs to strengthen faith-based sexual and reproductive health information provision.”* [**17 years old female discussant]**

“*Yes*, *it works*! *Because they are the leaders of the faith*. *They are active youths in all religions and will visit religious facilities*. *They are role models for others who are not in the religious facility*. *So*, *they can easily teach and can keep other young people from unwanted areas*.*”* **[30 years old Male, Key informant]**

“*Not only at the community level but also teaching and creating awareness in religious institutions is also acceptable to decrease the risk of exposure of youths to unnecessary activities*.*”* **[37 years old female, Key informant]**

### Subthemes: Strengthen community-based SRH services provision

According to most study participants, there is a need to prioritize strategies for strengthening community-based SRH services provision.

“*The government should educate adolescents about sexual and reproductive health issues starting from every kebele to make these services accessible.*”[**17 years old female discussant]**

“*If you want to teach this kind of education*, *you should teach young people by bringing them together rather than teaching them individually*. *So there should be a place for learning in a wide way so that everyone can use it*. *In our area*, *many young people want to learn such things in the kebele*.*”* [**18 years old female discussant]**

“*…*..*There is no platform that connects adolescents*, *parents*, *key community members*, *facility managers*, *service providers*, *health officers*, *higher officials*, *and researchers*. *If there is such a platform*, *we can solve easily any problems*. *This is the main problem I have seen in Ethiopia’s health system is this*. For such thing community-based platform is important”**[35 years Male, Key informant]**

## Discussion

Nowadays in Ethiopia, the mainstreaming of sexual and reproductive health services for adolescents has been increasing. Up-to-date evidence is required to understand and support national, regional, and local governments, and other stakeholders in the implementation of SRH policy and programs. Therefore, this study aimed to explore adolescents’ choices to obtain SRH services and their preferences for SRH services in public health facilities in Ethiopia. We found that public health facilities were not the preferred source of SRH services for adolescents, whereas, private pharmacies and traditional medical care were the preferred source of SRH services. We also found that the characteristics of SRH providers varied with the age of adolescents. While older adolescents favored service providers who were closer to their own age, younger adolescents favored those who were older. The neighborhood healthcare providers were not the preferred service providers for adolescents in this study.

The most preferred source of abortion services is traditional medical centers or traditionalists such as local herb sellers, herbalists, and traditional birth attendants [8]. There are perhaps multiple reasons for this, like confidentiality of services, trust of services provision, as well as accessibility and affordability of such services in their local area. This finding is in line with a previous study conducted in Western Nigeria [8]. This finding implies that the practice of alternative care for abortion care services seemed more common than seeking care in the health facility. Therefore, making abortion services accessible and affordability for adolescents is needed to save adolescents.

Adolescents prefer to access condoms and emergency pills from pharmacies rather than public health facilities. This finding is in line with the study conducted in Kenya, which shows that young people were successfully able to obtain ECP and condoms from pharmacies [38]. Similarly, Studies findings from Tanzania also support this study [39,40]. The finding highlights that co-designing pharmacy-based SRH services may be an effective strategy to improve access and utilization of services [41].

In this study, adolescents’ preferred service providers were not the local healthcare providers. Many participants said they would be willing to access SRH services from health workers they did not know and who live outside their community. Many participants were comfortable discussing their SRH issues with those providers who were not the local healthcare provider. This finding contradicts the previous study conducted in Western Nigeria [8], which shows that many participants would be willing to trust health workers they know who live within their community, and this is a neighborhood nurse promising approach [8]. However, this finding is in line with a previous study conducted in Western Kenya, which shows that the preferred service providers were not the local healthcare providers because they assumed they might not meet again and feel embarrassed about what they disclosed [24]. This might be due to those who are local healthcare providers breaking confidently. Most adolescents dislike such things. This finding implies that rotating SRH service providers from one ward to another may be an alternative approach.

The finding of this study found that the characteristics of SRH providers varied with age. While older adolescents favored service providers who were closer to their age, younger adolescents favored those who were older. This result is consistent with research findings from studies carried out in Western Kenya and Northwest Ethiopia [24,42]. The finding of this study found that male healthcare providers are preferred by adolescents to provide SRH services for them. Some others also preferred a same-sex preference for service providers. Female healthcare providers are less preferred for SRH services for adolescents due to behaviors like getting angry, hateful, and rumors to everyone. The results are consistent with research from the Awabel district in Northwest Ethiopia and Bhaktapur district in Nepal, which found that adolescents prefer same-sex service providers for SRH needs and are less likely to discuss SRH-related issues with opposite-sex providers [38,39]. However, this finding contradicts the finding of a study conducted in Bench-Sheko Zone; Southern Ethiopia revealed that most adolescents preferred to be treated by female healthcare providers [28].

### Conclusion

We conclude that public health facilities were not the preferred source of SRH services for adolescents. Confidentiality of services, trust in services provision, as well as availability and affordability of traditional medical care, make it preferred for adolescents. Private pharmacies are the preferred places to access condoms and emergency pills for adolescents because those places are less stressful compared to public health facilities. Neighborhood healthcare providers were not preferred for adolescents in this study, most adolescents are not comfortable discussing their SRH issues, they are afraid to ask and feel embarrassed. Male healthcare providers are preferred service providers for adolescents. Also, older adolescents preferred younger health workers and younger adolescents preferred older service providers. In addition, sexual partners, close friends, and peers who experienced similar problems are the preferred sources of SRH information for adolescents. Adolescents prefer pharmacies as a venue to access condoms and emergency pills, while they prefer traditional medical centers as a source of abortion services. Besides this, schools are a preferred venue for adolescents to access SRH information. Furthermore, study participants suggested the following strategies including engagement of private health clinics, engagement of faith-based organizations, and strengthening out of health facilities services provision. The study highlighted the need to consider the preferences of adolescents when designing their adolescent health programs. Besides this, the insight from this study may help to co-design adolescents’ responsive healthcare provision.

## Supporting information

S1 Appendix English version interview guide. "Supporting Information S1 Appendix: This file includes the English version of the interview guide utilized for conducting qualitative interviews with participants. It outlines the key questions and prompts intended to elicit in-depth responses aligned with the study objectives".
(DOCX)

S2 Appendix Participant demographics. "Supporting Information S2 Appendix: This file provides a comprehensive overview of participant demographics, detailing age, gender, and other relevant characteristics collected during the study".
(XLSX)
