# Supplementary material for: Exploring preferences to accessing sexual and reproductive health services: A qualitative study of adolescents’ and service provider perspectives
Source: PLoS One. 2024 Dec 4;19(12):e0312872. doi: 10.1371/journal.pone.0312872 (PMC11616883; doi:10.1371/journal.pone.0312872)
Supplement: S1 Appendix — "Supporting Information S1 Appendix: This file includes the English version of the interview guide utilized for conducting qualitative interviews with participants. It outlines the key questions and prompts intended to elicit in-depth responses aligned with the study objectives". (DOCX) [file pone.0312872.s001.docx]

**APPENDICES**

**APPENDIX 1A: English Version FGD Interview Guide**

|  | | |
| --- | --- | --- |
| **S.No.** | **Main Questions** | **Probing Questions** |
|  | In your community how do you see the need of adolescents for sexual and reproductive health services? (**Approachability)** | 1. *Please elaborate on what will do if adolescents have a sexual and reproductive health problem. Why?* 2. *Would you elaborate on how adolescents get information about sexual and reproductive health services?* 3. *Please elaborate on whether all adolescents have the right to access sexual and reproductive health services in this community?* |
|  | In your community how do you rate the **availability** of sexual and reproductive health services care for adolescents? (**Availability)** | 1. *Please elaborate on how easy or difficult for adolescents to access services when they want.* 2. *Would you please elaborate on why it was difficult or easy? (Please elaborate on how?)* 3. *Can adolescents get the services when they require them?* |
| 1. ` | Where do adolescents currently access sexual and reproductive health services in your area? | 1. *Please elaborate, on why some adolescents do not have access to sexual and reproductive health services?*   *(Is it due to a lack of information? Is it due to family-related issues? Is it due to social norms? Is it due to religion? Is it due to financial problems?)* |
|  | From where adolescents prefers to access sexual and reproductive health services easily and conveniently? | 1. *Please elaborate, why that place is preferred by adolescent?* |
|  | From whom adolescent prefers to access sexual and reproductive health services? | 1. *Please elaborate, about neighbourhood health professional?* 2. *Would you elaborate on the appropriate service delivery modality for adolescents?* 3. *Using digital health modality Community-based modality* 4. *Health facility-based modality* 5. *School-based modality* |
|  | With whom, adolescents comfortable to discussing their sexual and reproductive health concerns? | 1. *Please elaborate, their comfortably with their parents/caregivers, with their peers, with healthcare providers?* |

**APPENDIX 2A: Key Informant Interviews guide for health service providers**

| **S.No.** | **Main Questions** | **Probing Questions** |
| --- | --- | --- |
|  | How do you see the sexual and reproductive health services provision for adolescents? | 1. *Is there a system for adolescents to have knowledge about their own health and where and when to get health services?* 2. *Overall, what does your institution think about the provision of reproductive health services to young adults compared to adults?* 3. *Do you think that young adults are treated differently from adults? why? And why not?* 4. *Do you think that married and unmarried young people are treated differently? why? And why not?* |
|  | In your community how do you see the need of adolescents for sexual and reproductive health services? | 1. What are sexual and reproductive health services adolescents typically seek from local healthcare facilities? 2. *How do you see convenient operating hours, a welcoming and clean environment, and maintaining privacy and confidentiality?* 3. *Does it have the equipment, medicines, supplies, and technology needed to ensure effective service provision to adolescents?* |
|  | In your community **how do you rate** the accessibility of sexual and reproductive health services for adolescents? | 1. *How accessible are sexual and reproductive health services for adolescents?* 2. *Can they get it when they require it?* |
|  | From your point of view, do you think that sexual and reproductive health services are appropriate for adolescents? | 1. *How do you see the convenient your service is to adolescents?* 2. *How are your interactions with the adolescents?* 3. *Do adolescents participate in the provision of sexual and reproductive health services?* |
|  | From your point of view, from where and whom adolescent prefers to access sexual and reproductive health services? | 1. *Please elaborate, why that place is preferred by adolescent?* 2. *Please elaborate, why that provider is preferred by adolescent?* |
|  | How might we increase the accessibility of sexual and reproductive health services for adolescents so that they are able to seek out the services when they are needed? | 1. *Would you elaborate on the appropriate service delivery modality for adolescents?*  - *Using digital health modality Community-based modality* - *Health facility-based modality* - *School-based modality* |
